# Biocompatible N-acetyl-nanoconstruct alleviates lipopolysaccharide-induced acute lung injury in vivo

**DOI:** 10.1038/s41598-021-01624-5

**Published:** 2021-11-22

**Authors:** Seongchan Kim, Shin Young Kim, Seung Joon Rho, Seung Hoon Kim, So Hyang Song, Chi Hong Kim, Hyojin Lee, Sung Kyoung Kim

**Affiliations:** 1 Biomaterials Research Center, Biomedical Research Division, Korea Institute of Science and Technology (KIST), Seoul, Republic of Korea; 2 Division of Pulmonary and Critical Care Medicine, Department of Internal Medicine, St. Vincent’s Hospital, College of Medicine, The Catholic University of Korea, 93 Jungbu-daero, Paldal-gu, Suwon, Republic of Korea; 3 Research Institute of Medical Science, St. Vincent’s Hospital, College of Medicine, The Catholic University of Korea, Suwon, Republic of Korea

**Keywords:** Biotechnology, Medical research

## Abstract

Oxidative stress plays important roles in inflammatory responses during acute lung injury (ALI). Recently, nanoconstruct (Nano)-based drug-delivery systems have shown promise in many models of inflammation. In this study, we evaluated the anti-inflammatory effects of N-acetylcysteine (NAC) loaded in a biocompatible Nano using a rat model of ALI. We synthesized a Nano with a good NAC-releasing capacity using porous silica Nano, which was used to produce Nano/NAC complexes. For in vivo experiments, Sprague–Dawley rats were intraperitoneally administered NAC or Nano/NAC 30 min after intratracheal instillation of lipopolysaccharide. After 6 h, bronchoalveolar lavage fluids and lung tissues were collected. The anti-oxidative effect of the Nano/NAC complex was confirmed by demonstrating reduced levels of reactive oxygen species after treatment with the Nano/NAC in vitro. In vivo experiments also showed that the Nano/NAC treatment may protect against LPS‐induced ALI thorough anti‐oxidative and anti‐inflammatory effects, which may be attributed to the inactivation of the NF‐κB and MAPK pathways. In addition, the effects of Nano/NAC treatment were shown to be superior to those of NAC alone. We suggest the therapeutic potential of Nano/NAC treatment as an anti‐inflammatory agent against ALI. Furthermore, our study can provide basic data for developing nanotechnology-based pharmacotherapeutics for ALI.

## Introduction

Since the time acute respiratory distress syndrome (ARDS) was first introduced as a clinical entity in 1967^1^, many studies on ARDS have resulted in remarkable advances in the treatment and critical care management of patients with ARDS^2–4^. However, the mortality rate of ARDS remains very high (approximately 40%), and ARDS is one of the leading causes of mortality in intensive care units^5^. As the basic pathology of acute lung injury (ALI) and its severe form, ARDS, is inflammatory damage of the pulmonary endothelium and the alveolar epithelium, several pharmacotherapies based on these phenomena have been tested^6–8^. However, effective drugs for treating patients with ARDS have not been identified yet^9^.

Oxidative stress in the body results in an imbalance between reactive oxygen species (ROS) and anti-oxidants, causing cell or tissue damage^10^. Consequently, oxidative stress causes various diseases and is known to act as a pathogenic mechanism of ALI/ARDS^11,12^. N-acetylcysteine (NAC) is a precursor of glutathione (GSH), which is an endogenous anti-oxidant that prevents ROS-induced cell damage. NAC plays an important role in maintaining the oxidation–reduction balance of cells and in protecting cells from various intracellular and extracellular stimuli^13,14^. As NAC elicits an anti-oxidative effect during ROS-induced oxidative stress and inflammatory responses, several experiments based on NAC have been conducted using various inflammation models, including ALI^15–17^. Furthermore, based on these studies, some clinical studies have been conducted with patients with ARDS^18–20^. However, most of these clinical studies did not demonstrate therapeutic efficacy, and since the 2000s, few clinical studies have investigated the use of NAC in patients with ARDS. Several reasons may explain why NAC failed to prove effective in ARDS patients, and one of the potential causes relates to the pharmacological properties of NAC. NAC is hydrophilic and has a low bioavailability (4–10%), properties that inhibit the maintenance of an effective drug concentration in the blood and induce NAC excretion through the kidneys^21,22^. Therefore, new innovative approaches are required for improving the effectiveness of NAC.

Nanoconstructs (Nano) are an appropriate carrier for overcoming the physiological barriers and pharmacokinetics/pharmacodynamics limitations associated with traditional drug formulations and drugs alone^23^. Owing to the unique physical and chemical properties of Nano^24^, Nano-based drug-delivery systems provide distinct benefits by improving the pharmacokinetics/pharmacodynamics profiles of classical therapeutics and selectively delivering an effective dose of drugs to the desired site, thus reducing side effects. The effectiveness of Nano-based drug-delivery systems for treating various cancers has been demonstrated, and some of these approaches have been applied in clinical research^25^. With the development of nanotechnology, Nano can be employed in medical applications.

Here, we report the effect of NAC loaded in biocompatible Nano in a rat model of lipopolysaccharide (LPS)-induced ALI. To this end, we prepared porous silica Nano as a biocompatible platform, and developed an in vivo NAC-delivery system by introducing NAC inside the Nano, thus yielding “Nano/NAC” complexes. We expect that our findings indicate the potential role of Nano/NAC as an anti‐inflammatory agent in ALI, and following data could provide support for pharmacotherapeutic applications of Nano molecules against ALI.

## Methods

For full methods, please see the online supplement.

### Preparation of nano

Biocompatible Nano was prepared via sol–gel condensation and the surface was modified via silane chemistry. The product was characterized by nitrogen sorption and transmission electron microscopy (TEM) (see the online supplement for details).

### Animal experiments

All experimental protocols were approved by the Institutional Laboratory Animal Care and Ethical Committee of St. Vincent’s Hospital of The Catholic University of Korea. All methods were performed in accordance with the relevant guidelines and regulations and the Animal Research: Reporting of In Vivo Experiments guidelines (https://arriveguidelines.org/). Eight-ten-week-old male Sprague–Dawley rats were anesthetized and intubated with angiocatheter, followed by intratracheal instillation of 0.3 mL of LPS (3 mg/kg) (*Escherichia coli* O55:B5, Sigma-Aldrich) or saline through the angiocatheter. Rats were randomly assigned to the following 6 groups (n = 6 per group): (i) LPS group (subjected to LPS-induced ALI and injected with saline intraperitoneally 30 min after intratracheal LPS instillation); (ii) LPS + NAC group (subjected to LPS-induced ALI and injected with 200 mg NAC (dissolved in saline) intraperitoneally 30 min after intratracheal LPS instillation); (iii) LPS + Nano/NAC group (subjected to LPS-induced ALI and injected with Nano/NAC complex (200 mg NAC dissolved in Nano solution) intraperitoneally 30 min after intratracheal LPS instillation); (iv) Control group (intratracheally instilled with saline instead of LPS, and injected intraperitoneally with saline 30 min after intratracheal saline instillation); (v) NAC group (intratracheally instilled with saline instead of LPS, and injected intraperitoneally with 200 mg NAC 30 min after intratracheal saline instillation); and (vi) Nano/NAC group (intratracheally instilled with saline instead of LPS, and injected intraperitoneally with Nano/NAC 30 min after intratracheal saline instillation). At 6 h following intratracheal LPS or saline instillation, the rats were anesthetized and intubated again, and then bronchoalveolar lavage was performed. Thereafter, the rats were sacrificed to obtain lung tissues (see the online supplement for details).

### Measurements

Total cell counts, neutrophil counts, and cytokine levels (TNF-a and IL-6) in BALF were measured. Oxidative stress was analyzed by measuring intracellular ROS level in BALF and GSH/GSSG ratio and MDA and SOD activity in lung tissue homogenates. For histologic examination, lung tissues were stained with hematoxylin and eosin. The lung injury score was calculated using a scoring system^26^. All histological analyses were performed by an independent pathologist in a blinded fashion. Lung tissue homogenates were used for measuring MPO activity, for immunohistochemistry for iNOS, and for western blot for nuclear factor-kappa B (NF-kB) and mitogen-activated protein kinase (MAPK). In western blot analysis, we blotted the antibodies after cutting the membrane to the appropriate size for each antibody using a protein ladder marker, so we have no images of adequate length (see the online supplement for details).

### Bio-TEM for nano

The excised lungs from the rats were frozen in situ, followed by processing for electron microscopy based on the protocol of the National Instrumentation Center for Environmental Management at Seoul National University.

### Statistical analysis

All statistical analyses were performed using the GraphPad Prism 8 software. Data are presented as means ± standard error. Differences between groups were analyzed using one-way ANOVA followed by a Bonferroni post hoc test for multiple comparisons. *P* < 0.05 was considered statistically significant.

## Results

### Intracellular uptake of loaded, biocompatible, and non-toxic Nano/NAC in vitro

**Figure 1:**
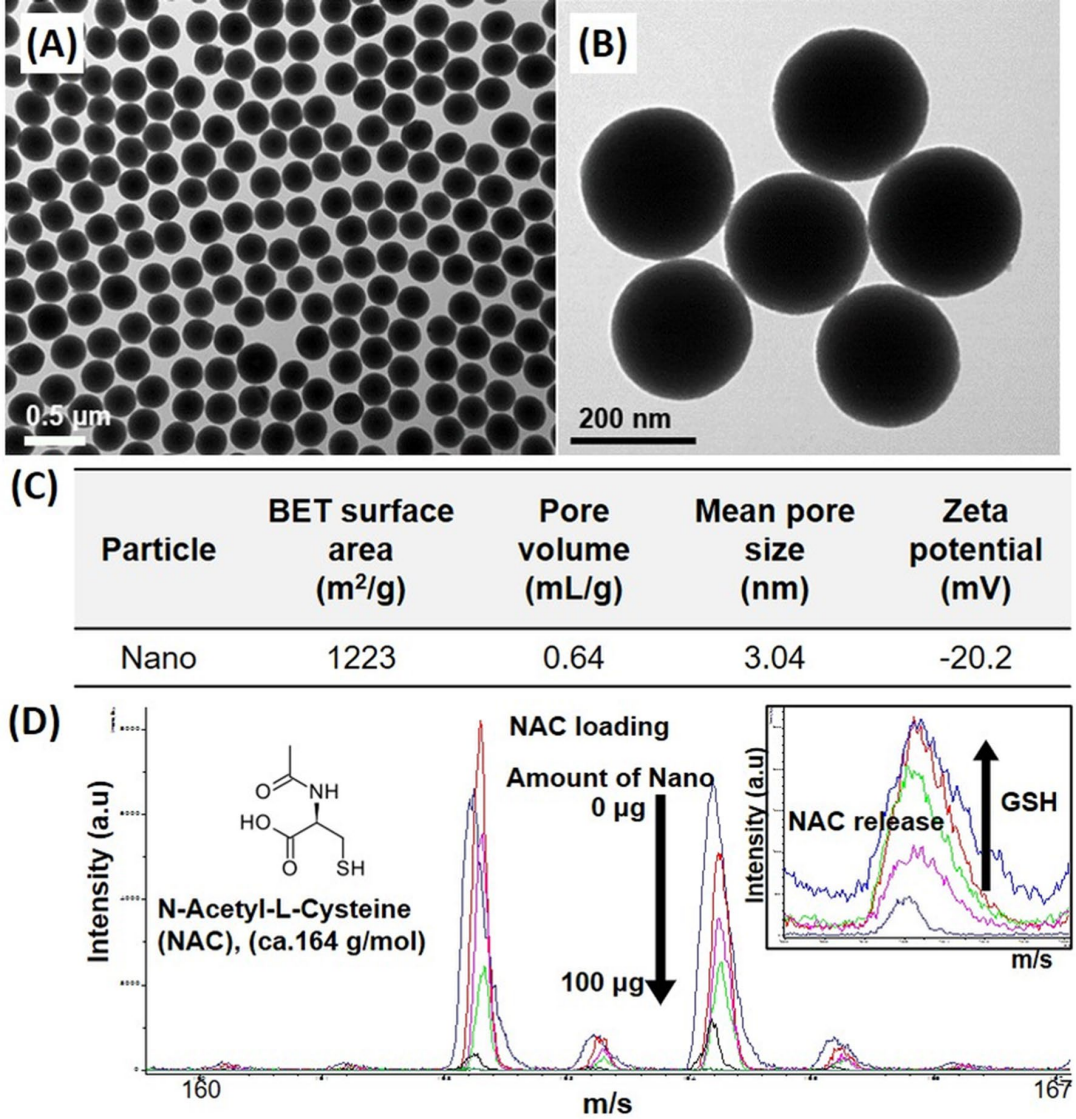
Characterization of the Nano developed in this study. (**A**, **B**) Transmission electron microscope images of Nano. The images showed that the Nano had a monodispersed morphology with a diameter of approximately 200 nm. (**C**) Nitrogen absorption/desorption analysis was performed to analyze the Brunauer–Emmett–Teller surface area, pore volume, and mean pore size of the Nano. Based on the result of the nitrogen sorption and zeta potential analysis, the prepared Nano possessed a pore size of ~ 3 nm with a cavity and a negative surface charge (-20.2 mV), which was sufficient to load NAC inside the pore. (**D**) Mass spectrometry analysis of the NAC loading and release profile. To investigate the controlled release of NAC under reducing conditions, we measured the NAC-release profiles in the presence of various GSH concentrations (0–10 mM) by mass spectrometry. The results showed that 75% of NAC was released within 1 h, confirming the GSH-dependent release kinetics of NAC from Nano/NAC. *Nano* Nanoconstruct, *NAC* N-acetylcysteine, *GSH* Reduced glutathione.

We first prepared a functional Nano as a vehicle for NAC delivery. To enable successful loading and controllable release of NAC, the prepared Nano was further functionalized with surface thiol groups. Transmission electron microscopy (TEM) images showed that the Nano had a monodispersed morphology with a diameter of approximately 200 nm (Fig. 1A,B). Nitrogen sorption and zeta potential analyses revealed that the prepared Nano possessed a pore size of ~ 3 nm, with an internal cavity and a negative surface charge (− 20.2 mV) (Fig. 1C), which was sufficient to load NAC inside the pores. The grafted thiol groups (used for introducing NAC) were previously verified^27^.

After simply mixing NAC with the Nano in phosphate-buffered saline for 30 min at room temperature (thereby generating Nano/NAC complexes), the NAC-loading capacity of the Nano was evaluated using mass spectroscopy, which revealed 50% (w/w) loading capacity (Fig. 1D). To investigate the controlled release of NAC through the cleavage of disulfide bonds under reducing conditions, we measured the release profiles of NAC under various GSH concentrations (0–10 mM) by mass spectrometry. The results showed that 75% of the NAC was released over a course of 1 h (inset of Fig. 1D). Therefore, we confirmed the GSH-dependent release kinetics of NAC from Nano/NAC.

Prior to performing in vivo experiments, we examined the biocompatibility and intracellular-uptake efficiency. We measured the viability of human A549 cells (adenocarcinomic alveolar basal epithelial cells) after treating them with various concentrations of Nano; > 90% of the cells were viable after treatment with Nano at all concentrations tested (see Fig. S1 in the online data supplement). To evaluate the intracellular uptake of Nano, they were labeled with tetramethylrhodamine (TRITC-Nano). After treating the A549 cells with Nano, the cells were visualized by fluorescence microscopy (see Fig. S2 in the online data supplement). Fluorescence corresponding to Nano was observed in the cytoplasm, indicating that the Nano successfully localized inside the cells. These results demonstrated the biocompatibility of Nano and its potential use for delivering NAC.

### Effects of Nano/NAC on ROS production in LPS-induced inflammation in vitro

**Figure 2:**
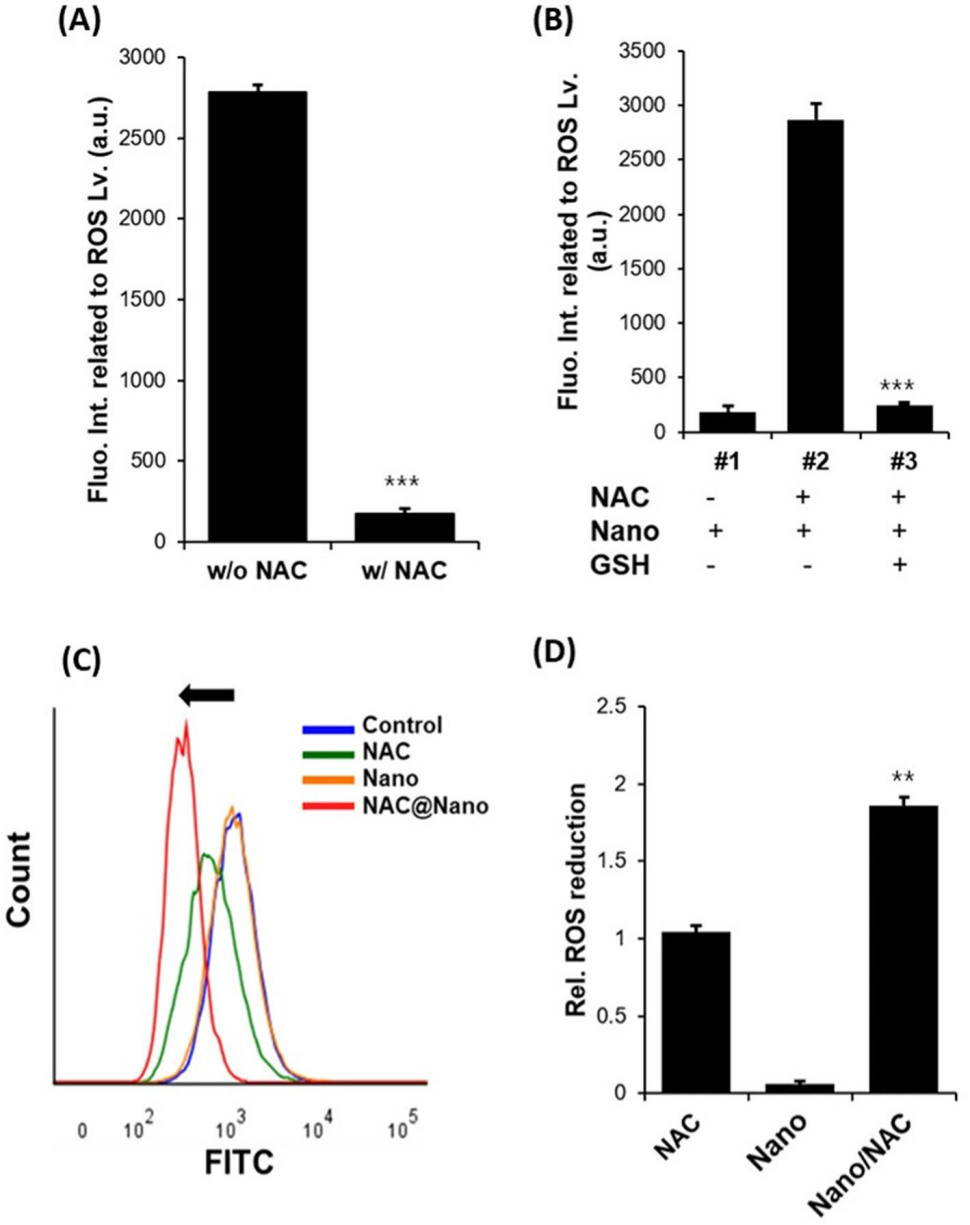
Effects of Nano/NAC on ROS levels in vitro. (**A**, **B**) Detecting fluorescence intensities to measure ROS levels in vitro. NAC was effective in reducing ROS levels. Moreover, ROS levels significantly decreased in the presence of GSH, indicating the selective release of NAC from Nano/NAC and a subsequent decrease of ROS levels. (**C**, **D**) Flow cytometric analysis of ROS reduction in live cells. The intensity of 2′,7′-dichlorofluorescein fluorescence (formed after oxidization by ROS) in the Nano/NAC-treated group was significantly lower than that observed in the free NAC- and free Nano-treated groups, indicating that Nano/NAC alleviated oxidative stress by efficiently delivering NAC into cells, followed by the selective release of NAC in the presence of reductants. *Nano* Nanoconstruct, *NAC* N-acetylcysteine, *ROS* Reactive oxygen species, *GSH* Reduced glutathione.

To investigate the anti-oxidative effect of Nano/NAC, we preliminarily verified the ROS-scavenger ability of NAC. As hydrogen peroxide (H_2_O_2_) is the main species driving oxidative stress^28^, we examined the ability of NAC to reduce H_2_O_2_-related ROS levels. Figure 2A shows the ROS-reduction efficacy of NAC. ROS levels significantly decreased in the presence of GSH (10 mM), indicating the selective release of NAC from Nano/NAC and the subsequent decrease in ROS levels (Fig. 2B).

Next, we investigated the effectiveness of Nano/NAC at inhibiting ROS production in vitro. A549 cells were incubated with Nano/NAC (loaded with 0.5 mM NAC), free NAC (0.5 mM), and free Nano for 24 h after incubation with LPS, after which the intracellular ROS levels were measured by 6-carboxy-2′,7′-dichlorodihydrofluorescein diacetate assays. The fluorescence intensity was significantly lower in the Nano/NAC-treated group than in the free NAC- and free Nano-treated groups, due to the ROS-dependent oxidation of carboxy-2′,7′-dichlorodihydrofluorescein diacetate, resulting in the production of a non-fluorescent molecule, 2′,7′-dichlorofluorescein (Fig. 2C). The bar graph in Fig. 2D indicates that Nano/NAC alleviated oxidative stress through efficient intracellular NAC delivery with subsequent release of NAC into the cellular environment. This result suggested that our Nano-based NAC-delivery strategy may enable protection against LPS‐induced ALI via anti‐oxidative effects.

### Effects of Nano/NAC on inflammatory cells and pro-inflammatory cytokines in LPS-induced inflammation in vivo

**Figure 3:**
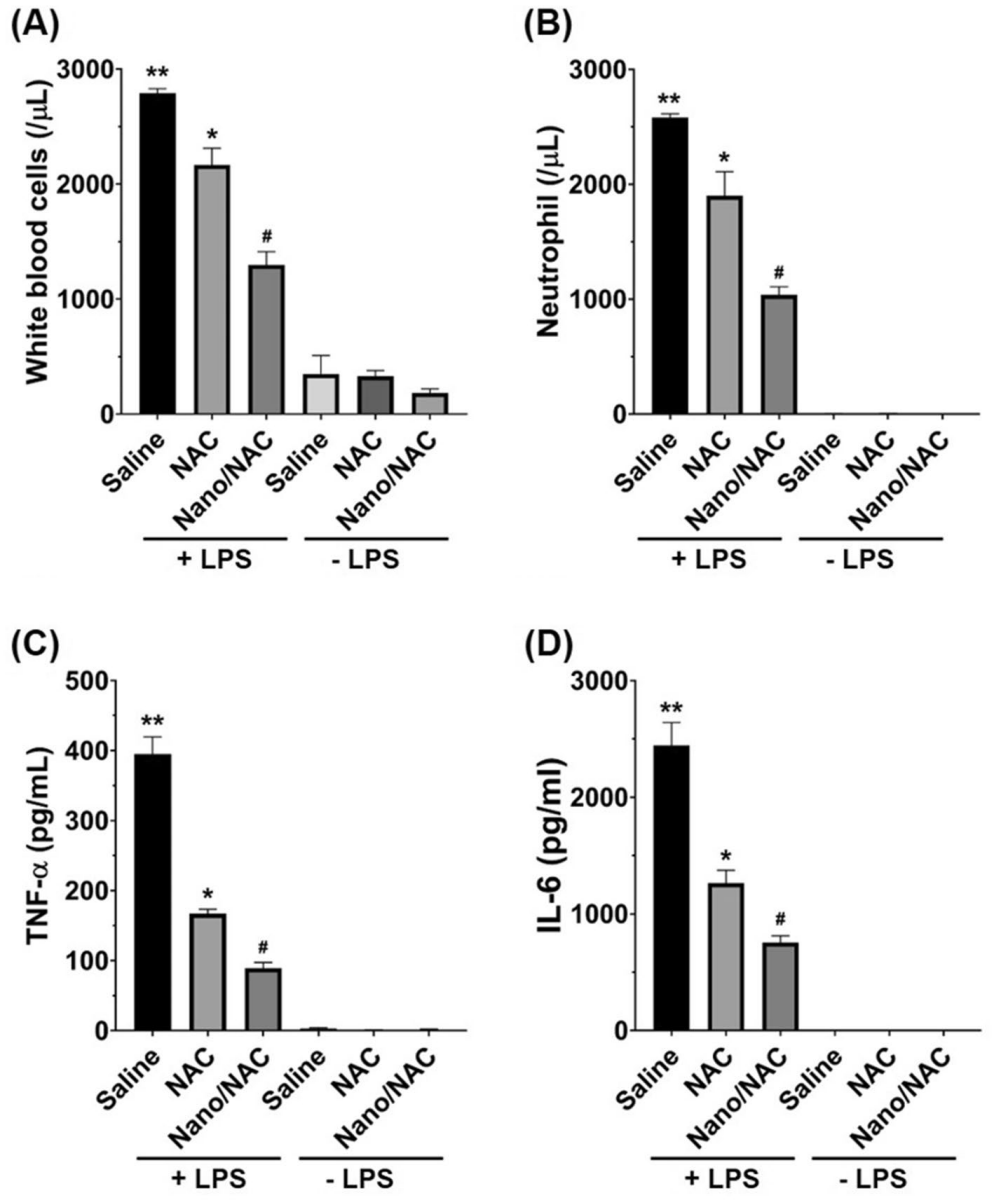
Effects of Nano/NAC on inflammatory cell counts and cytokine levels in BALF. (**A**, **B**) Total cell and neutrophil counts in BALF. (**C**, **D**) TNF-α and IL-6 levels in BALF. The LPS group showed significantly higher cell counts and inflammatory cytokine levels than the control group. The LPS + NAC group showed significantly lower cell counts and inflammatory cytokine levels than the LPS group. The LPS + Nano/NAC group showed significantly lower cell counts and inflammatory cytokine levels than the LPS + NAC group. The data shown are expressed as the mean ± standard error (SE; n = 6 rats/group). **P < 0.05 versus the control group; *P < 0.05 versus the LPS group; #*P* < 0.05 versus the LPS + NAC group. *Nano* Nanoconstruct, *NAC* N-acetylcysteine, *BALF* Bronchoalveolar lavage fluid, *LPS* Lipopolysaccharide.

Cell counts and inflammatory cytokine levels (including TNF-α and IL-6) in BALF were assessed to determine the anti-inflammatory activities of NAC and Nano/NAC. The numbers of total cells and neutrophils, as well as the TNF-α and IL-6 levels, in BALF were significantly higher in the LPS-treated group. The LPS + NAC and LPS + Nano/NAC groups exhibited significantly lower total cell numbers, neutrophil counts, and TNF-α and IL-6 levels in BALF, than did the LPS group. However, these parameters decreased significantly more in the LPS + Nano/NAC group than in the LPS + NAC group. No differences were observed in the cell counts and inflammatory cytokine levels in BALF between the control and NAC or Nano/NAC group (Fig. 3).

### Effects of Nano/NAC on oxidative stress in LPS-induced inflammation in vivo

**Figure 4:**
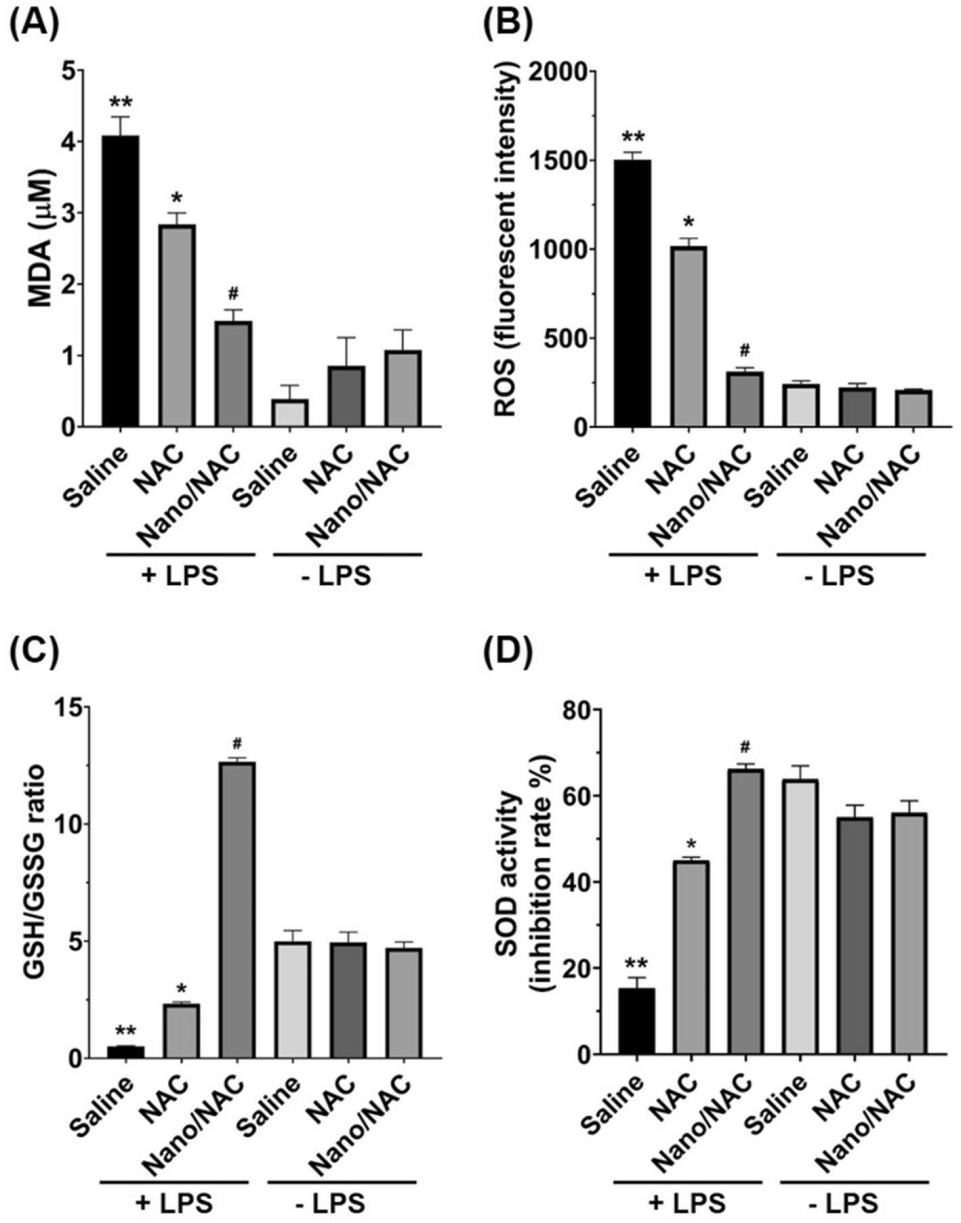
Effects of Nano/NAC on MDA contents in BALF and the intracellular ROS levels, GSH/GSSG ratios, and SOD activities in lung tissues. (**A**, **B**) The LPS group showed significantly higher MDA contents and ROS levels than the control group. The LPS + NAC group showed significantly lower MDA contents and ROS levels than the LPS group. The LPS + Nano/NAC group showed significantly lower MDA contents and ROS levels than the LPS + NAC group. (**C**, **D**) The LPS group showed significantly lower GSH/GSSG ratios and SOD activities than the control group. The LPS + NAC group showed significantly higher GSH/GSSG ratios and SOD activities than the LPS group. The LPS + Nano/NAC group showed significantly higher GSH/GSSG ratios and SOD activities than the LPS + NAC group. The data shown are expressed as the mean ± SE (n = 6 rats/group). **P < 0.05 versus the control group; *P < 0.05 versus the LPS group; #*P* < 0.05 versus the LPS + NAC group. *Nano* Nanoconstruct, *NAC* N-acetylcysteine, *MDA* Malondialdehyde, *BALF* Bronchoalveolar lavage fluid, *ROS* Reactive oxygen species, *GSH* Reduced glutathione, *GSSG* Oxidized glutathione, *SOD* Superoxide dismutase, *LPS* Lipopolysaccharide.

ROS levels and MDA contents were significantly higher in the LPS group. The LPS + NAC and LPS + Nano/NAC groups showed significantly reduced ROS and MDA contents, compared with the LPS group. However, these contents decreased significantly more in the LPS + Nano/NAC group than in the LPS + NAC group. The GSH/GSSG ratio and SOD activity decreased significantly in the LPS group. The LPS + NAC and LPS + Nano/NAC groups exhibited significantly increased GSH/GSSG ratios and SOD activities compared with the LPS group. However, these parameters increased significantly higher in the LPS + Nano/NAC group than in the LPS + NAC group (Fig. 4).

### Effects of Nano/NAC on LPS-induced lung pathology in vivo

**Figure 5:**
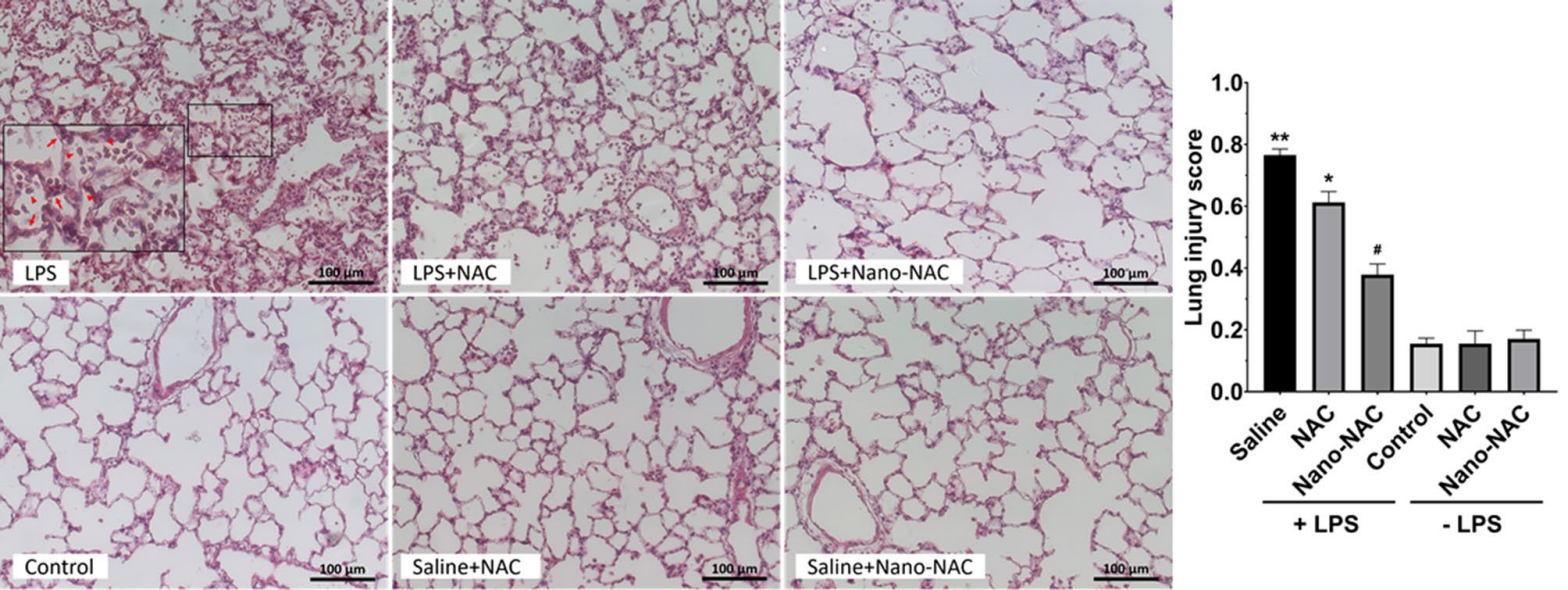
Effects of Nano/NAC on histological alterations in lung tissues. The lung tissues from the LPS group demonstrated significant pathological changes, including alveolar and interstitial neutrophil infiltration (arrow head in magnified box), alveolar and interstitial hemorrhage, alveolar septal thickening (arrow in magnified box), and proteinaceous debris in the alveoli, when compared with the control group. These pathological changes in the LPS group were significantly attenuated by co-treatment with NAC or Nano/NAC, with a greater significant difference observed in the Nano/NAC group (hematoxylin and eosin staining, × 200). The lung-injury score, an objective measure of the degree of ALI, was significantly higher in the LPS group than in the control group. The lung-injury score was significantly decreased by co-treatment with NAC or Nano/NAC, with a greater significant difference observed in the Nano/NAC group. No significant difference was found between the control and NAC or Nano/NAC groups in terms of the lung-injury scores. The data shown are expressed as the mean ± SE (n = 6 rats/group). **P < 0.05 versus the control group; *P < 0.05 versus the LPS group; #*P* < 0.05 versus the LPS + NAC group. *Nano* Nanoconstruct, *NAC* N-acetylcysteine, *ALI* Acute lung injury, *LPS* Lipopolysaccharide.

As shown in Fig. 5, the lung tissues in the LPS group demonstrated significant pathological changes, including alveolar and interstitial neutrophil infiltration, alveolar and interstitial hemorrhaging, alveolar septal thickening, and proteinaceous debris in the alveoli. These pathological changes induced by LPS were markedly attenuated by further treatment with NAC or Nano/NAC, especially in the Nano/NAC group, which showed significant differences. The lung-injury score, an objective measure of the degree of ALI, significantly increased in the LPS group compared with that in the control group. The lung-injury score was significantly decreased by NAC or Nano/NAC, with a significant difference being observed in the Nano/NAC group (Fig. 5). No significant differences in lung-injury scores were observed between the control and NAC or Nano/NAC groups.

### Effects of Nano/NAC on MPO activity in LPS-induced inflammation in vivo

**Figure 6:**
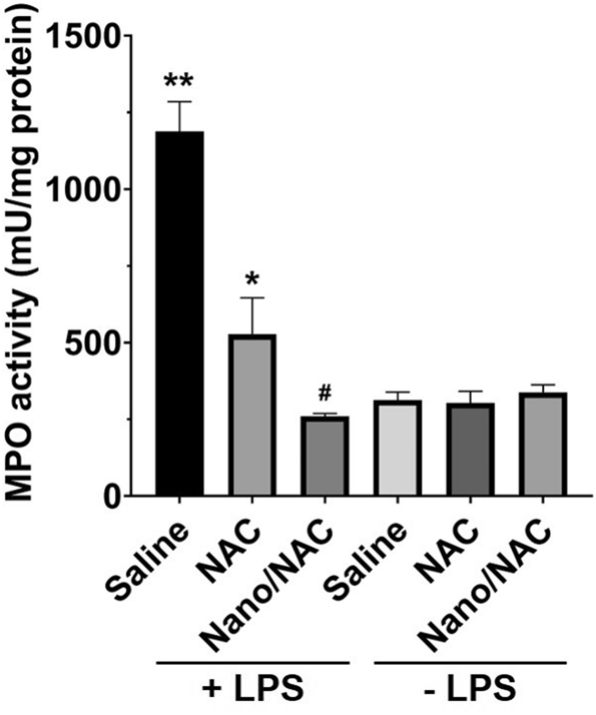
Effects of Nano/NAC on MPO activities in lung tissues. The LPS group exhibited significantly higher MPO activities than the control group. The LPS + NAC group showed significantly lower MPO activities than the LPS group. The LPS + Nano/NAC group showed significantly lower MPO activities than the LPS + NAC group. The data shown are expressed as the mean ± SE (n = 6 rats/group). **P < 0.05 versus the control group; *P < 0.05 versus the LPS group; #*P* < 0.05 versus the LPS + NAC group. *Nano* Nanoconstruct, *NAC* N-acetylcysteine, *MPO* Myeloperoxidase, *LPS* Lipopolysaccharide.

The increased infiltration of inflammatory cells in lung tissues observed upon hematoxylin and eosin staining (Fig. 5) was associated with an influx of leukocytes into the lung tissues. Therefore, we investigated the effect of NAC or Nano/NAC on neutrophil infiltration by measuring MPO activity. The MPO activity increased significantly in the LPS group and was significantly attenuated by co-treatment with NAC or Nano/NAC. However, the MPO activity was reduced more significantly by Nano/NAC than by NAC (Fig. 6).

### Effects of Nano/NAC on iNOS expression in LPS-induced inflammation in vivo

**Figure 7:**
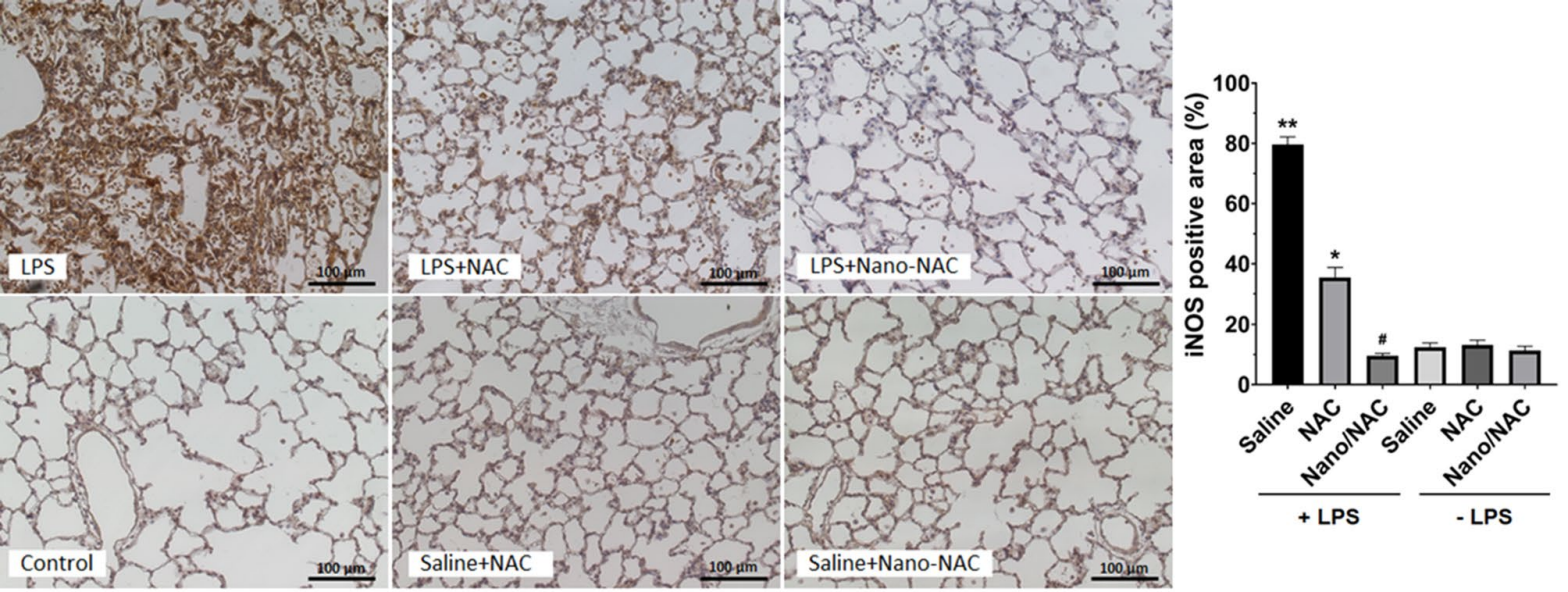
Effects of Nano/NAC on iNOS expression in lung tissues. Lung sections obtained from the control group showed minimal iNOS staining. In contrast, lung sections from the LPS group showed significantly higher iNOS staining than those derived from the control group. The increased iNOS staining in the LPS group was markedly attenuated by NAC or Nano/NAC co-treatment, with a greater significant difference observed in the Nano/NAC group (immunohistochemical staining for iNOS, × 200). Quantitative analysis showed that iNOS expression was significantly higher in the LPS group than in the control group. However, iNOS expression was significantly decreased by NAC or Nano/NAC co-treatment, with a greater significant difference observed in the Nano/NAC group. The data shown are expressed as the mean ± SE (n = 6 rats/group). **P < 0.05 versus the control group; *P < 0.05 versus the LPS group; #*P* < 0.05 versus the LPS + NAC group. *Nano* Nanoconstruct, *NAC* N-acetylcysteine, *iNOS* Inducible nitric oxide synthase, *LPS* Lipopolysaccharide.

The control group demonstrated minimal iNOS expression. Significantly increased iNOS expression in lung tissues was observed in the LPS group, compared with that in the control group. LPS plus NAC or Nano/NAC treatment markedly inhibited iNOS expression, compared with LPS treatment alone, with a greater significant difference observed in the LPS + Nano/NAC group. Quantitative analysis confirmed that iNOS expression was significantly increased in the LPS group, compared with that in the control group. However, iNOS expression was significantly decreased by NAC or Nano/NAC, with a greater significant decrease caused by Nano/NAC. No significant difference was observed between the control and NAC or Nano/NAC groups in terms of iNOS expression (Fig. 7).

### Effects of Nano/NAC on the activation of NF-kB and MAPK in LPS-induced inflammation in vivo

**Figure 8:**
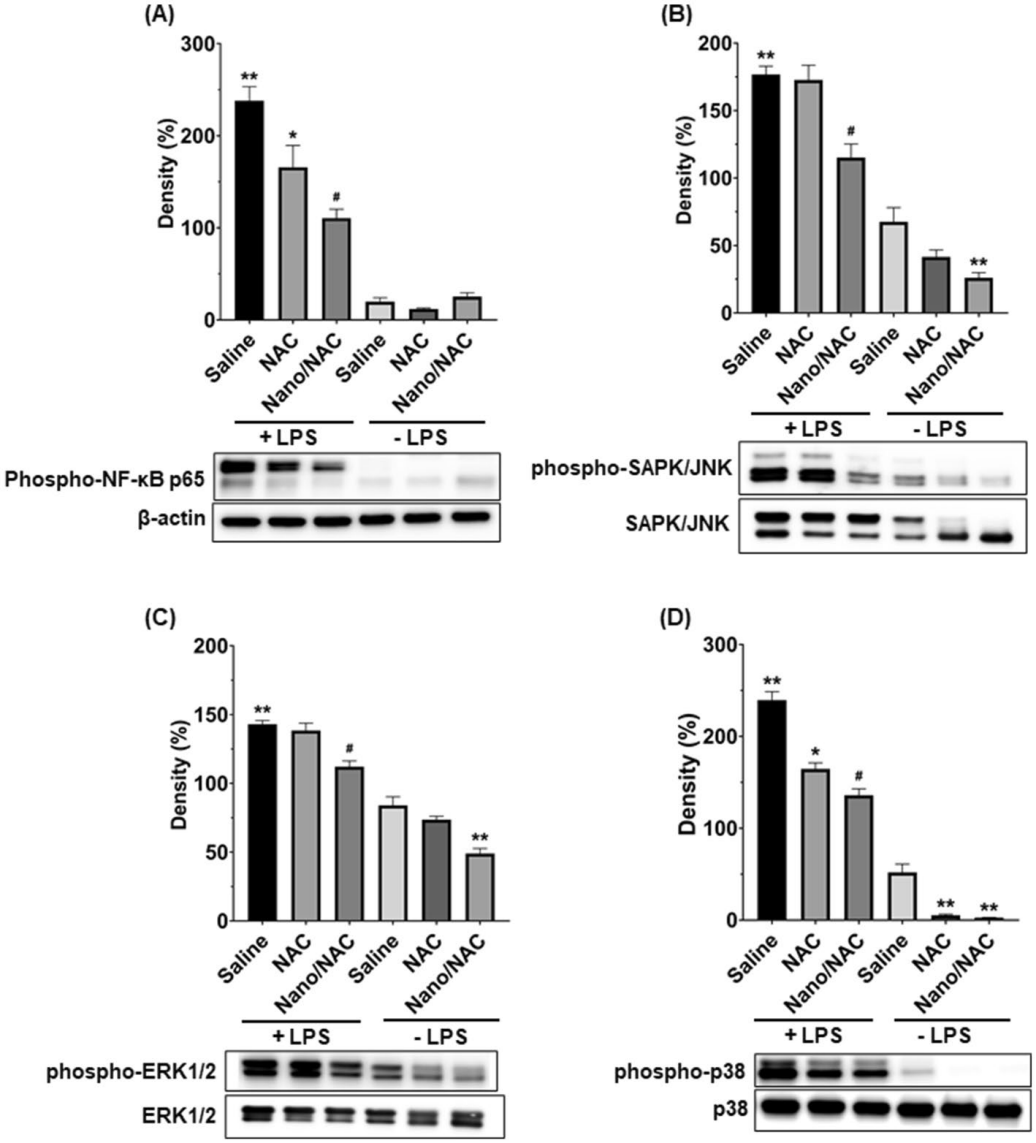
Effects of Nano/NAC on activation of the NF-kB and MAPK pathways in lung tissues, as determined by western blot analysis. Expression of β-actin, SAPK/JNK, ERK1/2, and p38 was used as a loading control. (**A**) LPS treatment elicited a significant increase in the level of phospho-NF-κB p65. NAC or Nano/NAC treatment significantly reduced the level of phospho-NF-κB p65, with a greater significant difference observed after Nano/NAC co-treatment. (**B**–**D**) Significant increases in the phospho-SAPK/JNK, phospho-ERK1/2, and phospho-p38 levels were observed in the LPS group. NAC treatment significantly suppressed the levels of phospho-p38, but not those of phospho-SAPK/JNK and phopho-ERK1/2. However, Nano/NAC treatment significantly suppressed the levels of phospho-SAPK/JNK, phospho-ERK1/2, and phospho-p38. The level of phospho-p38 was decreased significantly more by Nano/NAC treatment than by NAC treatment (**D**). **P < 0.05 versus the control group; *P < 0.05 versus the LPS group; #*P* < 0.05 versus the LPS + NAC group. *Nano* Nanoconstruct, *NAC* N-acetylcysteine, *LPS* Lipopolysaccharide.

We evaluated NF-κB p65 activation by western blotting to explore the mechanism underlying the attenuation of ALI with NAC or Nano/NAC. As shown in Fig. 8A, LPS elicited a significant increase in the level of phospho-NF-κB p65. NAC or Nano/NAC significantly reduced the level of phospho-NF-κB p65, with a greater significant decrease being observed after Nano/NAC treatment. Activation of the MAPK-signaling pathway plays a crucial role in regulating inflammation. Therefore, we performed western blotting to investigate the activation (phosphorylation) of SAPK/JNK, ERK1/2, and p38 in lung tissue homogenates. Significant increases in the levels of phospho-SAPK/JNK, phospho-ERK1/2, and phospho-p38 were observed in the LPS group. NAC significantly suppressed the levels of phospho-p38, but not those of phospho-SAPK/JNK and phopho-ERK1/2 (Fig. 8B,C). However, Nano/NAC significantly suppressed the levels of phospho-SAPK/JNK, phospho-ERK1/2, and phospho-p38. The level of phospho-p38 was more significantly decreased by Nano/NAC than by NAC (Fig. 8D).

## Discussion

In this study, we synthesized a Nano with a good NAC-releasing capacity using porous silica Nano, which was used to produce Nano/NAC complexes. By demonstrating reduced ROS levels after treatment with the Nano/NAC complex in vitro, the anti-oxidative effect of the Nano/NAC complex was confirmed. In vivo experiments also showed that the Nano/NAC complex had anti-oxidative and anti-inflammatory effects in the rat model of LPS-induced ALI, which may have been due to inhibition of the NF-kB and MAPK pathways. Furthermore, the anti-oxidative and anti-inflammatory effects of the Nano/NAC complex were superior to those of NAC alone.

ALI is an acute inflammatory pulmonary disease that is characterized by clinical, radiological, and physiological abnormalities due to increased inflammation and permeability of alveolar-capillary membrane resulting from various causes^29^. Its severe form is known as ARDS^30^. Although the pathogenic mechanism whereby ALI leads to ARDS development has not been clearly identified, it is believed that different types of cells, mediators, and humoral elements interact in a complex manner, causing damage to vascular endothelial cells and alveolar epithelial cells, resulting in increased permeability of the alveolar capillary membrane in the lungs, eventually leading to the development of non-cardiac pulmonary edema^31^. ARDS is characterized by the infiltration of inflammatory cells, especially activated neutrophils. These activated neutrophils induce cellular or tissue damage by releasing cytotoxic and pro-inflammatory agents, such as proteolytic enzymes, pro-inflammatory cytokines, lipid mediators, and ROS. Excessive ROS induced by activated neutrophils play key roles in lung injury and ARDS progression^11,12^. In turn, elevated ROS levels induce the production of pro-inflammatory cytokines or adhesion molecules to further promote lung damage and pulmonary edema. These effects perpetuate a destructive loop by attracting more inflammatory cells that in turn generate additional cytotoxic materials, eventually contributing to severe injury of the alveolar-capillary membrane and acute respiratory failure. In other words, oxidative stress can trigger dysfunction of the microvascular endothelium and alveolar epithelium, culminating in massive neutrophil infiltration followed by the secretion of pro-inflammatory mediators. Therefore, preventing excessive ROS production (i.e., reducing oxidative stress) can potentially serve as an approach for treating ARDS.

In this respect, several studies in the ALI model employing various anti-oxidants have been conducted, and their anti-inflammatory effects have been confirmed^32,33^. However, clinical trials unfortunately did not show consistently positive results^34,35^. Several reasons may explain the lack of efficacy in the clinical trials. First, the pathophysiology of ARDS is heterogenous^30,31^. As various cells, cytokines, and signaling pathways are involved, it is difficult to expect effective treatment with drugs that only inhibit one mechanism. In addition, multiple-organ dysfunction can affect drug-specific pharmacokinetics/pharmacodynamics profiles, which may reduce the effectiveness of drugs^36,37^. To overcome this issue, many studies have recently been conducted with the aim of developing drug-delivery systems, based on nanotechnology^38,39^. Using nanotechnology, the release and absorption of drugs can be effectively controlled, and the side effects of drugs can be reduced, while maximizing efficacy; thus, the necessary amount of drug can be effectively localized to the target site for a certain period of time. We therefore investigated the effect of the anti-oxidant, NAC, using nanotechnology. Our results showed that the Nano/NAC complex had superior anti-oxidative and anti-inflammatory effects when compared to NAC alone.

In this study, NAC treatment alone did not effectively inhibit the SAPK/JNK and ERK1/2 pathways, consistent with several previous studies reporting that NAC itself exhibits anti-inflammatory effects by inhibiting p38 without affecting the SAPK/JNK and ERK1/2 pathways^40,41^. Although NAC was somewhat effective, it is possible that the overall effect was diminished because it did not act on any MAPK pathways. This possibility may explain why NAC was not very effective for patients with ARDS, characterized by heterogeneous pathophysiology and multiple organ dysfunction. However, we were able to overcome this problem by loading NAC into Nano and administering the Nano/NAC complex in a rat model of LPS-induced ALI, which induced significant suppression of all three MAPK pathways studied.

Previous studies have confirmed the effectiveness of various Nano as drug-delivery vehicles, using different ALI models. In several studies, phospholipid nanomicelles were used to confirm the anti-inflammatory effects of drugs in an ALI model^42,43^. Another group generated a hybrid peptide-gold Nano-based TLR inhibitor that inhibited toll-like receptors and showed an anti-inflammatory effect in an ALI model. In particular, they showed that the anti-inflammatory effect was dependent on the size of the Nano^44,45^. Yu et al. developed a lipid-based nanocarrier and confirmed the anti-inflammatory effect of oleic acid in their ALI model. They also showed that the anti-inflammatory effect of the Nano was size-dependent^46^. As such, nanomedicine can be used to develop various kinds of Nano, which can be used to administer drugs. In this study, we prepared a porous silica Nano and strategically applied it for our experiments. The loading and release of NAC were controlled via porosity and chemical surface modification of Nano (see Fig. S3 in the online data supplement). Moreover, because of its large hydrodynamic size in serum, the porous silica Nano mainly accumulated in the lungs^47^. In addition, Yu et al. reported high accumulation of a porous silica Nano in the lungs through an in vivo biodistribution and pharmacokinetics study^48^. We therefore monitored this phenomenon in the current study, as shown in Fig. S4 in the online data supplement. The data showing desirable cellular localization of Nano and controlled delivery of drug successfully guaranteed pharmacological efficiency in vivo.

Although the porous silica Nano exhibited advantages, such as high porosity, structural stability, and ease of surface functionalization^49,50^, biosafety issues related to the porous silica Nano itself must be considered before employing it in clinical practice. However, porous silica Nano have exhibited excellent biosafety and bioavailability with low cellular toxicity in several previous studies^47,48,51,52^. In addition, silica is an endogenous substance in the human body, especially in the bones, and is often used in tablet-form drug formulations as an excipient, which supports the safety and stability of silica as a generally-recognized-as-safe substance by the United States Food and Drug Administration^53,54^. Recently, a diagnostic application of an amorphous silica Nano received FDA approval in a phase-I clinical trial, suggesting that porous silica Nano is an ideal candidate for drug-delivery systems^55^.

In conclusion, we demonstrated that Nano/NAC treatment may protect against LPS‐induced ALI thorough anti‐oxidative and anti‐inflammatory effects, which may be attributed to the inactivation of the NF‐κB and MAPK pathways. In addition, the effects of Nano/NAC treatment were shown to be superior to those of NAC alone. We suggest the therapeutic potential of Nano/NAC treatment as an anti‐inflammatory agent against ALI. Furthermore, our study can provide basic data for developing nanotechnology-based pharmacotherapeutics for ALI or ARDS.

## Supplementary Information

Supplementary Information.
