# Supplementary material for: Biocompatible N-acetyl-nanoconstruct alleviates lipopolysaccharide-induced acute lung injury in vivo
Source: Sci Rep. 2021 Nov 22;11:22662. doi: 10.1038/s41598-021-01624-5 (PMC8608841; doi:10.1038/s41598-021-01624-5)

**Online supplementary materials:**

Supplementary methods

Supplementary figures

Supplementary reference

Original western blot images

**Supplementary methods**

Materials

Cetyl trimethyl ammonium bromide (CTAB) was purchased from Acros (New Jersey, USA). Tetramethyl orthosilicate (TMOS), toluene, dimethylsulfoxide (DMSO), (3-Mercaptopropyl)trimethoxysilanewere (MPTES) purchased from Sigma-Aldrich, USA. PBS (Phosphate buffered saline), FBS (Fetal bovine serum), and P/S (penicillin and streptomycin) were purchased from Welgene, Korea. CCK-8 assay kit was purchased from Dojindo Molecular Technologies, Inc, USA.

Preparation and Characterization of Nano

To prepare the monodisperse-sized biocompatible Nano, tetramethyl orthosilicate (TMOS) solution was added to the solution by using a tube connected syringe-pump at 0.5 mL/h into the 4 g of Cetyl trimethyl ammonium bromide (CTAB) dissolved in a basic solution (1M NaOH), followed by stirring for 6 h and aging overnight without stirring. The synthesized product was centrifuged and washed with ethanol and water. For surface modification, the Nano was suspended in the (3-Mercaptopropyl)trimethoxysilanewere (MPTES) solution, and the suspension was refluxed at 120℃ overnight, washed with ethanol and water. The pore size, surface area, and pore volume of the Nano were analyzed through nitrogen sorption experiments. To remove the CTAB, 12M of HCl was added to Nano in ethanol and refluxed at 120℃ overnight. Nitrogen sorption isotherms were obtained using a NOVA Surface Area Analyzer (Nova 2200e, Quantachrome instrument). Before the measurements, the sample was degassed for 12 h at 573 K. The morphological study was carried out using transmission electron microscopy (TEM, JEM1010, JEOL) and zeta potential was measured by zetasizer NS90 (Malvern).

Measurement of reactive oxygen species *in vitro*

ROS assay was assessed according to a manufacturer's instructions using Carboxy-H_2_DCFDA (Ca-H) Cellular ROS Assay Kit (Abcam) and the fluorescence intensity of 2',7'-dichlorofluorescein (DCF), an oxidized form of Ca-H by ROS, was measured. First, an H_2_O_2_ (30%) standard curve was obtained and used to determine H_2_O_2_ concentration in samples. To measure the ROS reduction ability of NAC, 0.5 mM of NAC is mixed with H_2_O_2_ solution for 2 h at RT, followed by ROS assay using Ca-H. To verify the ROS reduction activity of Nano/NAC, The Nano/NAC complex incubated with H_2_O_2_ solutions, and then GSH was added to the complex for 2 h at RT, followed by ROS assay using Ca-H.

To measure intracellular ROS index *in vitro*, A549 cells (3×10^4^ cells/well, 24-well plate) were pre-treated with LPS (1 μg/mL) for 24 h to generate ROS condition (reference S2-S4), and the cells were incubated with free NAC, Nano, and Nano/NAC for 24 h. The cells were incubated with certain amount of Ca-H for 45 min and washed twice with PBS to remove excess chemical reagent. The cells were harvested with trypsin-EDTA, and immediately analyzed by Flow cytometry and microplate reader (Beckton Dickinson).

Cell culture

A549 (human adenocarcinomic alveolar basal epithelial cell) was cultured in RPMI-1640 (Welgene) supplemented with 10% FBS and 1% P/S in 37 ℃ and 5% CO_2_.

Cell viability assay

To measure the cell viability of Nano for biological application, A549 cells (1×10^4^ cells/well) were prepared in a 96-well plate in triplicate for 24 h, followed by incubation with various concentrations of Nano with complete medium. The cells were then carefully washed with PBS, then CCK-8 cell proliferation assay solution was added for 1 h, followed by measuring absorbance at 450 and 670 nm with reduction calculation by using a microplate reader (Molecular Devices, Inc.).

Cellular imaging

To verify whether the Nano localize into the cells, Tetramethylrhodamine (TRITC)-labelled Nano were prepared, then A549 cells (2×10^4^ cells/well, 24-well plate) were treated with the complex for 4 h. After the cells were stained with Hoechst 33342 for nucleus staining, fluorescence images were monitored with an inverted fluorescence microscope (Olympus).

Animals

We purchased 8- to 10-week-old male Sprague-Dawley rats from Coatech, Korea, and kept them in the animal facility. Two rats were housed in each cage and allowed to acclimate for 1 week prior to treatment. The cages were kept at room temperature with an alternating 12-h light/dark cycle, and the animals were supplied with regular rodent chow and water ad libitum. The research was approved by the Institutional Laboratory Animal Care and Ethical Committee of St. Vincent’s Hospital of The Catholic University of Korea and carried out in compliance with the Animal Research: Reporting of In Vivo Experiments guidelines (https://arriveguidelines.org/).

LPS-induced ALI model

Anesthesia was induced with 4% isoflurane in 100% oxygen at 6 L/min flow rate using 2 L, transparent plastic chambers. Isoflurane mixed with oxygen was administered using an isoflurane vaporizer (Harvard Apparatus). Adequate anesthesia depth was measured by loss of toe pinch reflex. The rat was placed on a clean, disinfected tilting work stand (Hallowell EMC) and suspended by a rubber band hooked around the upper incisors under continuous anesthesia sustained by isoflurane through nasal cone. The animal’s mouth was opened with a cotton tip, and the tongue was moved to one side. The vocal cords were visualized by inserting the otoscope into the oral cavity while pushing the tip of the epiglottis forward with the tip of the otoscope. Under direct vision of the upper airways, a 14-gauge angiocatheter was inserted orally through the vocal cords via the inside of the otoscope cone while the vocal cords were visualized. The rats were treated intratracheally with 0.3 mL of LPS (3 mg/kg) (Escherichia coli O55:B5, Sigma-Aldrich) through angiocatheter with a microsyringe.

Experimental groups

Rats were randomly assigned to the following 6 groups: (i) LPS group. Rats were subjected to LPS-induced ALI and injected with saline intraperitoneally at 30 min after intratracheal LPS instillation (n=6). (ii) LPS+NAC group. Rats were subjected to LPS-induced ALI and injected with 200 mg NAC (dissolved in saline) intraperitoneally at 30 min after intratracheal LPS instillation (n=6). (iii) LPS+Nano/NAC group. Rats were subjected to LPS-induced ALI and injected with Nano/NAC complex (200 mg NAC dissolved in Nano solution) intraperitoneally at 30 min after intratracheal LPS instillation (n=6). (iv) Control group. Saline was instilled intratracheally instead of LPS, and it was injected intraperitoneally instead of NAC or Nano/NAC at 30 min after intratracheal saline instillation (n=6). (v) NAC group. Saline was instilled intratracheally instead of LPS, and 200 mg NAC was injected intraperitoneally at 30 min after intratracheal saline instillation (n=6). (vi) Nano/NAC group. Saline was instilled intratracheally instead of LPS, Nano/NAC was injected intraperitoneally at 30 min after intratracheal saline instillation (n=6).

Collection of BALF and lung tissue

At 6 h following intratracheal LPS or saline instillation, the rats were anesthetized and intubated as indicated above. Bronchoalveolar lavage was performed with 5 mL of saline via angiocatheter. Each lavage was performed 3 times and the lavage fluid was obtained (except first lavage fluid). The obtained BALF was moved to a 15 mL falcon tube packed in ice. Half of the obtained BALF was prepared for intracellular ROS and cell count, and the remainder was preserved at -70°C before the cytokine concentration was measured. After bronchoalveolar lavage, the rats were exsanguinated via the abdominal aorta. A median thoracotomy was performed and the chest wall was opened. For the measurement of malondialdehyde (MDA) content, GSH/GSSG ratio, superoxide dismutase (SOD) activity, myeloperoxidase (MPO) activity, inducible nitric oxide synthase (iNOS) expression, and Western blot analysis, the right lung was removed and stored immediately at -70°C until use. The left lung was extracted for histology and lung injury scoring.

Measurement of cell count and cytokines in BAL fluid

After centrifugation of BALF at 2,000 g for 10 min at 4°C, the supernatant was discarded. After removing the supernatant, the sediment was rinsed twice with RPMI 1640. Cell count was measured by hemocytometer. The total cell count was expressed as the number of cells per mL of BALF. After adjusting the total cell number to 1.0 × 10^6^/mL, 200 µL of the sample was centrifuged, smeared on a glass slide, air-dried, and then stained with Wright-Giemsa. A differential count was obtained by counting 500 cells in a high-power view (Ⅹ 400) light microscopic field using ocular lens with a grid. BALF collected for ELISA was pooled and centrifuged at 2,000 g for 10 min at 4°C. The supernatant was discarded and assayed immediately. TNF-α and IL-6 levels in the supernatant were determined using an ELISA kit (R&D systems) following the manufacturer’s manual.

Measurement of oxidative stress

The intracellular level of ROS was measured using 2',7'-dichlorofluorescein diacetate (DCFH-DA; Sigma-Aldrich). Cell pellets obtained after centrifugation of BALF were washed with phosphate buffered saline (PBS) and incubated with 20 μM DCFH-DA for 10 min at 37˚C. The intracellular ROS activity was then detected by fluorescence measurement at 488 nm excitation and 525 nm emission on a fluorescence plate reader. Lung tissue samples were homogenized in PBS as 10% (w/v). The homogenates were centrifuged (2,000 g, 10 min, 4°C), and the supernatant was used for assay of GSH/GSSG ratio and MDA and SOD activity by following the manufacturer’s protocol. GSH/GSSG ratio was calculated by using the equation in the manual (Abcam) after measuring GSH and total GSH concentration of the homogenates by monitoring fluorescence at 490 nm. MDA, the end product of lipid peroxidation, was determined by measuring thiobarbituric acid reactive substances (TBARS) using TBARS assay kit (Cayman Chemical). The absorbance was determined at 532 nm and expressed as nmol/gram of tissue. In addition, the SOD-liked activity was determined by colorimetric method using a SOD determination kit (Sigma-Aldrich). The absorbance was recorded at 450 nm and the SOD-liked activity (inhibition rate %) levels were calculated by using the equation in manufacturer’s protocol.

MPO activity

Neutrophil sequestration in the lungs was analyzed by measuring the activity of tissue MPO. The MPO activity was assessed using MPO activity assay kit (Sigma-Aldrich) according to the manufacturer’s protocol. Tissue samples were thawed, homogenized in 20 mM phosphate buffer (pH 7.4), and centrifuged (13,000 g, 10 min, 4°C). The pellet was resuspended in 50 mM phosphate buffer (pH 6.0) containing 0.5% CTAB. The suspension was subjected to four cycles of freezing and thawing and further disrupted by sonication for 40 s. The sample was centrifuged again, and the supernatant was assayed for MPO activity using kinetic readings for 3 min. The absorbance was measured at 460 nm, and the results were presented as changes in optical density/min/gram lung tissue at 460 nm.

Histological examination and lung injury score

Lung tissue samples were fixed in 10% (w/v) PBS-buffered formaldehyde for 1 week and 4-μm-thick sections were prepared from paraffin-embedded tissues. Sections were deparaffinized with xylene, stained with hematoxylin and eosin. The lung injury score was calculated using a scoring system reported in ‘An Official American Thoracic Society Workshop Report: Features and Measurements of Experimental Acute Lung Injury in Animals’ (reference S1). As noted in the report, we selected five independent variables that are hallmarks of the ALI: alveolar neutrophils, interstitial neutrophils, hyaline membranes, proteinaceous debris filling the airspaces, and alveolar septal thickening. To produce the lung injury score, the sum of each of the five variables was weighed according to the relevance associated to each feature of ALI, and then was standardized to the number of fields assessed. All the histological analyses were performed by an independent pathologist in a blinded fashion.

Immunohistochemical examination

Immunohistochemistry was performed using the paraffin-embedded formalin-fixed lung tissues. After deparaffinization and hydration, endogenous peroxidase was quenched with 3% (v/v) hydrogen peroxide in 60% (v/v) methanol for 30 min. Blocking was performed with 1.5% goat serum, and after appropriate washing, the sections were incubated with iNOS antibody (1:100, Sigma-Aldrich) at 4°C overnight. After incubation with the primary antibody, sections were washed with PBS twice and incubated with anti-rabbit secondary antibody (Vector Laboratories) for 1h, washed with PBS twice and developed with DAB (Vector Laboratories). Images were taken with BX51 (Olympus) with same magnification (X 10). To quantify the expression of iNOS, images of stained cells were analyzed using Image J software (NIH). The acquired images in RGB color were digitally separated into different color channels of hematoxylin and diaminobenzidine (DAB) by the color deconvolution tool. The extent of staining was calculated as the DAB-positive area divided by the hematoxylin-positive area. The iNOS expression was calculated as the DAB-positive area divided by the total tissue area.

Western blotting analysis for NF-kB and MAPK

Lung tissue was homogenized with a PRO-PREP (Intron bio). The homogenates were incubated for 30 min on ice and centrifuged (12,000 g, 5 min, 4°C). The supernatant was collected and stored at -70°C until use. The total protein concentration in the extract was determined using a Bradford protein assay. The total protein (20 μg) was resolved on a 10% SDS-polyacrylamide pre-cast gel and transferred to polyvinylidene difluoride membrane. The membrane was blocked for 1 h at room temperature with a blocking solution. The blot was incubated overnight at 4°C with antibodies against phospho-NF-κB p65 (1:1000, Santa Cruz Biotechnology), ERK1/2, phospho-ERK1/2, SAPK/JNK, phospho-SAPK/JNK, p38 and phospho-p38 (1:1000, Cell Signaling). After three washing steps, the membrane was incubated with a secondary antibody (1:1000, Cell Signaling) for 1 h at room temperature. The signals were detected using Amersham ECL Prime (GE Healthcare), and band intensities were quantified Image J software (NIH). For the quantification of western blot analysis, the ratio of phosphorylation of NF-kB p65 to beta actin and phosphorylation to total form of MAPK was determined. Expression of β-actin, SAPK/JNK, ERK1/2, and p38 was used as a loading control.

**Supplementary figures**


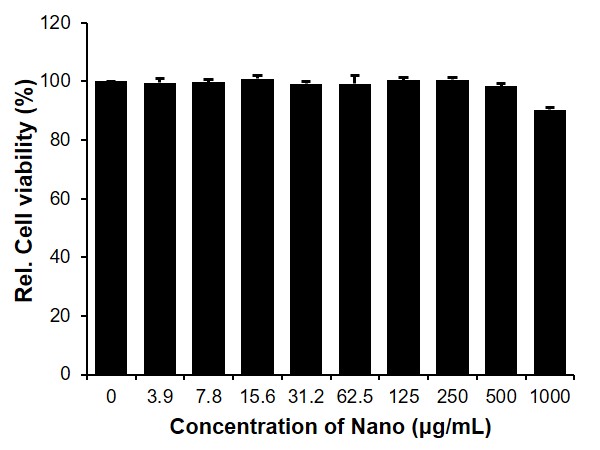


Figure S1. Cell viability test. More than 90% of the cells were viable under all concentrations of Nano.


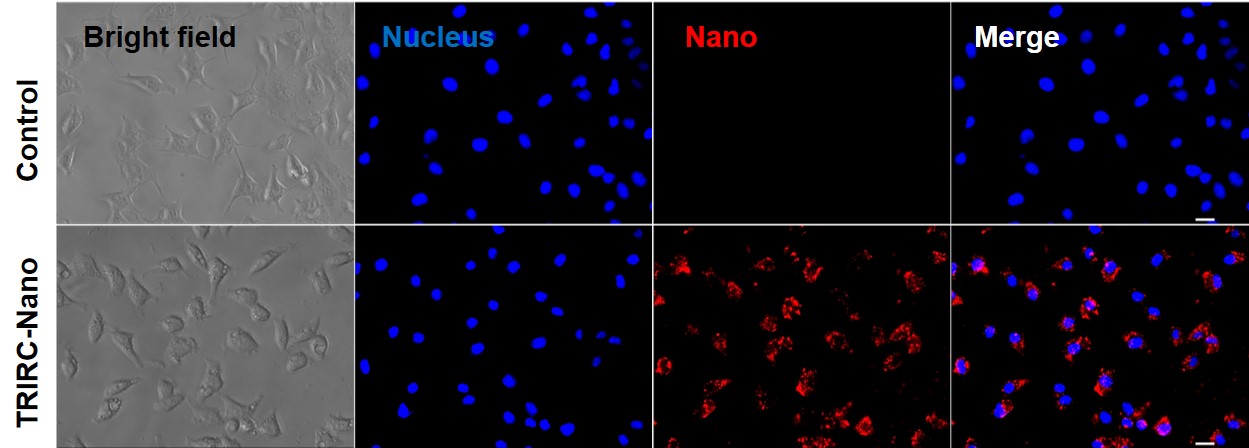


Figure S2. Intracellular uptake study. Fluorescence corresponding to Nano observed in cytoplasm, indicating the successful localization inside the cells.


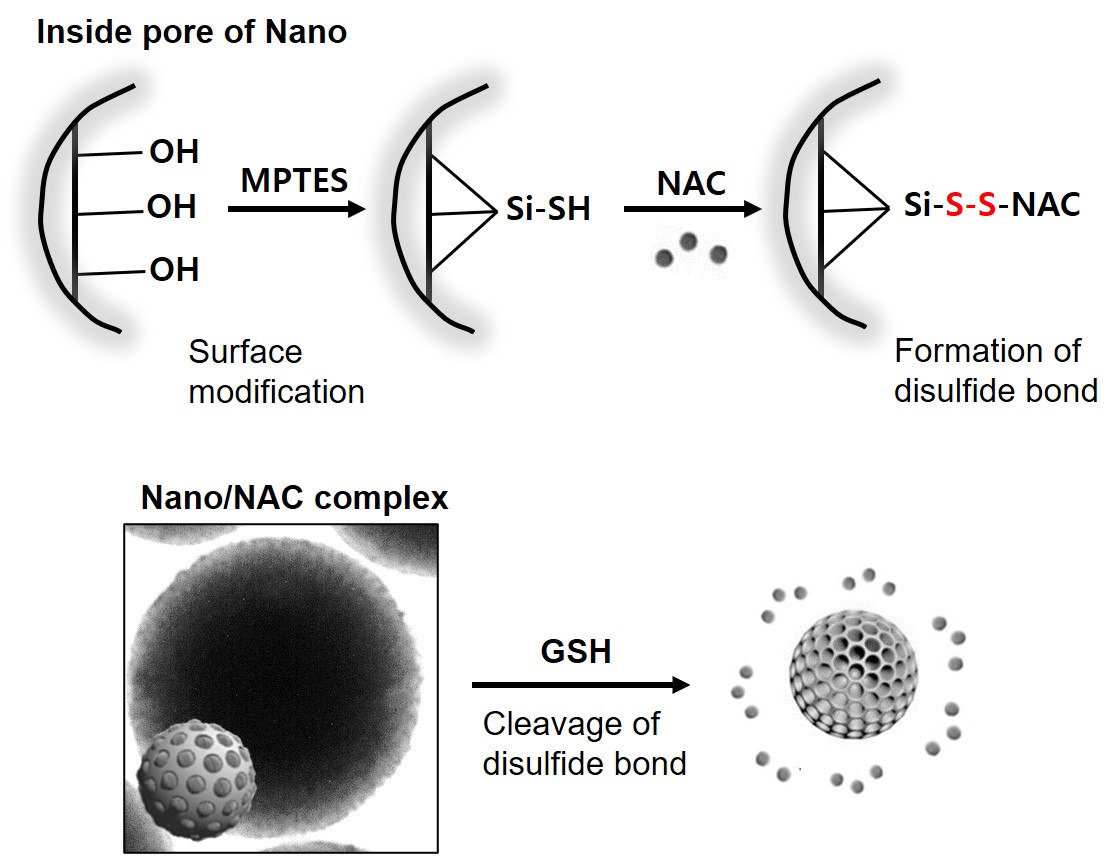


Figure S3. The loading and release of NAC were sophisticatedly controlled via porosity and chemical surface modification of Nano. Images were created using Autodesk 3D MAX 2017 (https://www.autodesk.co.kr/) and Microsoft PowerPoint 2016 (https://www.microsoft.com/ko-kr/microsoft-365/microsoft-office?rtc=1)


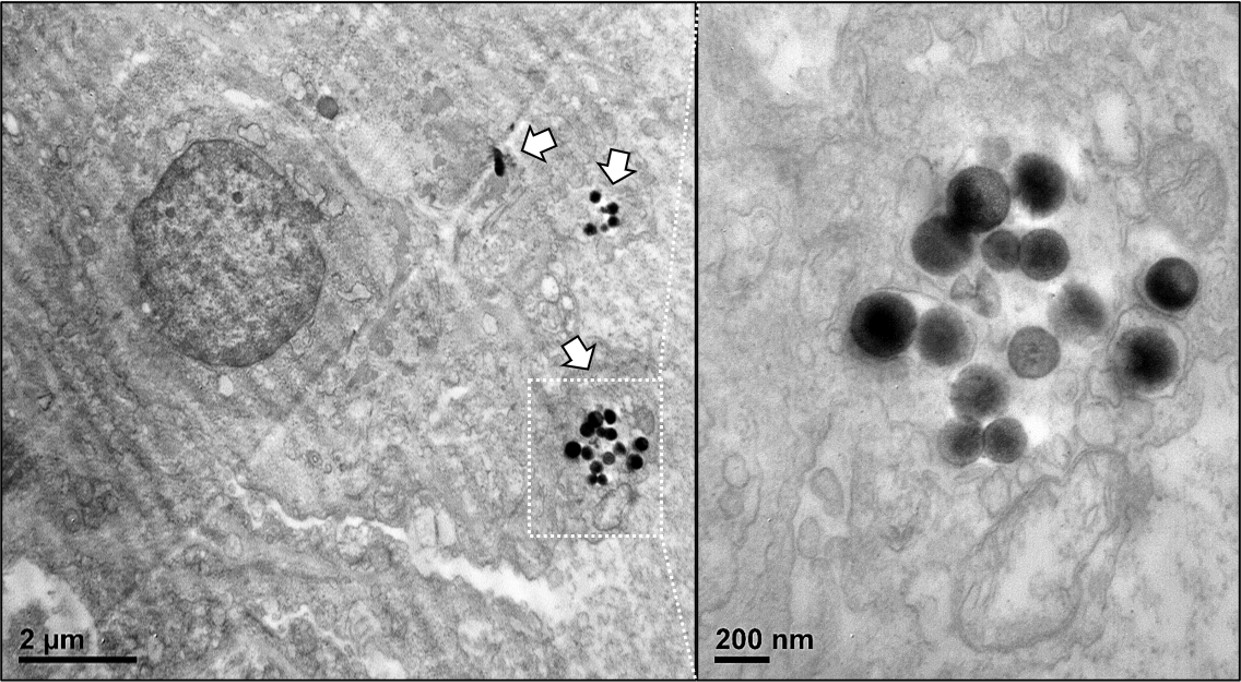
Figure S4. Bio-TEM images of lung tissue sections following Nano/NAC treatment. The left image shows the presence of internalized Nano (white arrows) inside the cytoplasm.

**Supplementary reference**

S1. Matute-Bello G., *et al.* An official American Thoracic Society workshop report: features and measurements of experimental acute lung injury in animals. *Am J Respir Cell Mol Biol* **44**, 725-738 (2011).

S2. Wu Y. *et al*., Activation of TLR4 Is Required for the Synergistic Induction of Dual Oxidase 2 and Dual Oxidase A2 by IFN-g and Lipopolysaccharide in Human Pancreatic Cancer Cell Lines, *The Journal of Immunology*, **190**, 1859-1872 (2013).

S3. Roux C. *et al.*, Reactive oxygen species modulate macrophage immunosuppressive phenotype through the up-regulation of PD-L1, *Proc. Natl. Acad. Sci. U. S. A*, **116**, 4326-4335 (2019).

S4. Gao F. *et al.*, Curcumin alleviates LPS-induced inflammation and oxidative stress in mouse microglial BV2 cells by targeting miR-137-3p/NeuroD1, *RSC Adv*., **9**, 38397-38406 (2019).

**Original western blot images**


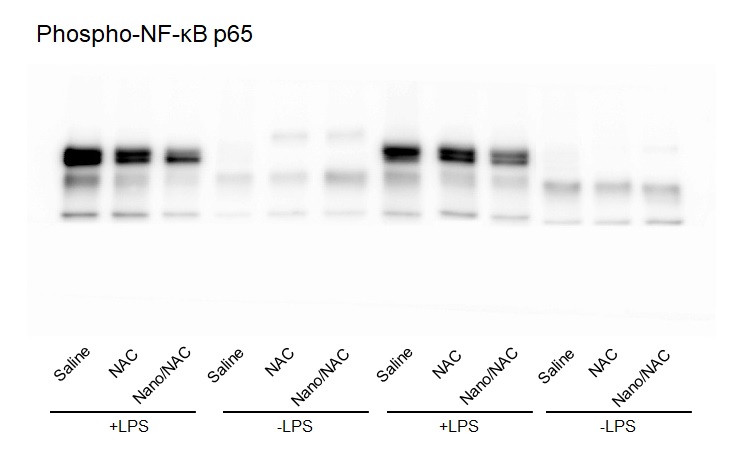


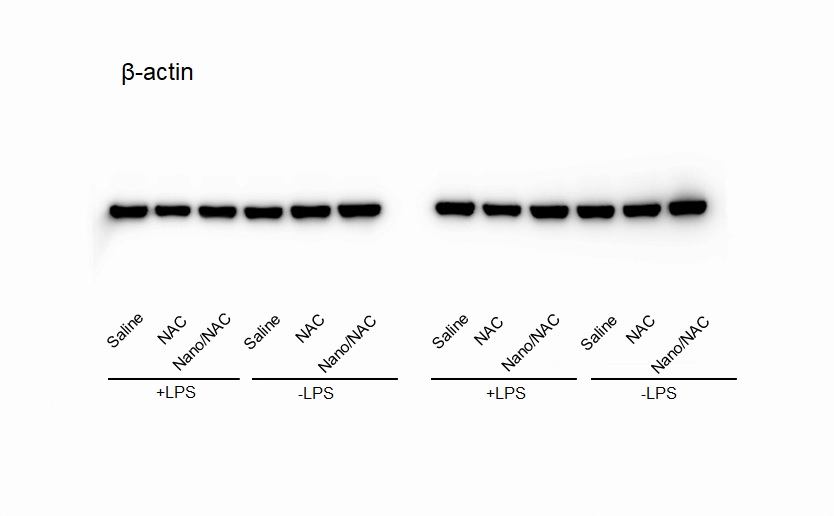


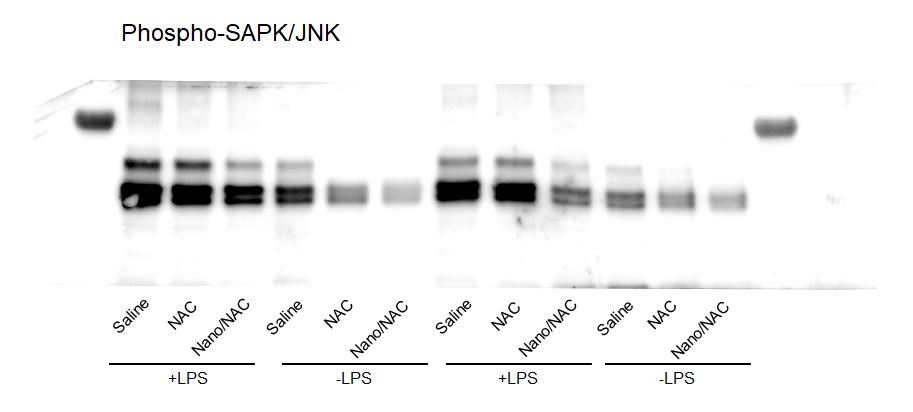


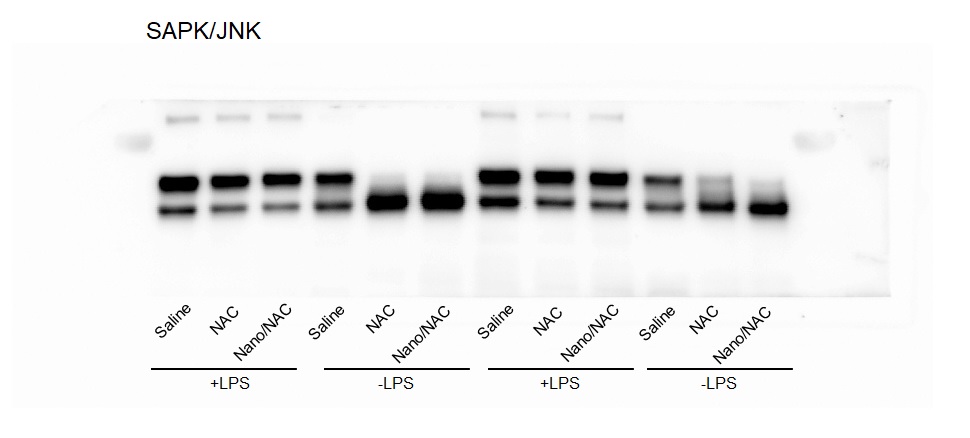


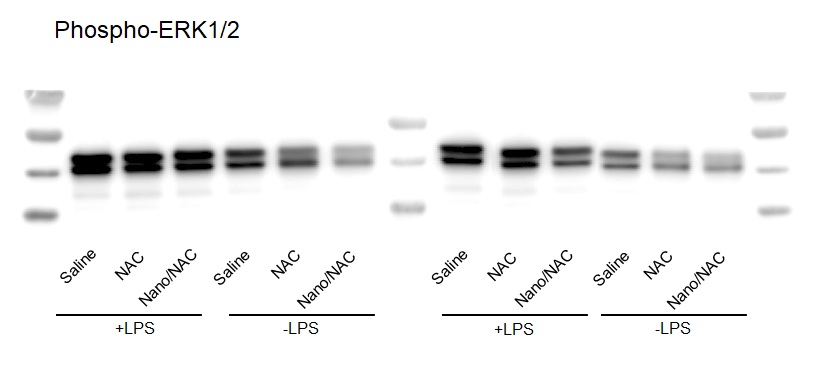


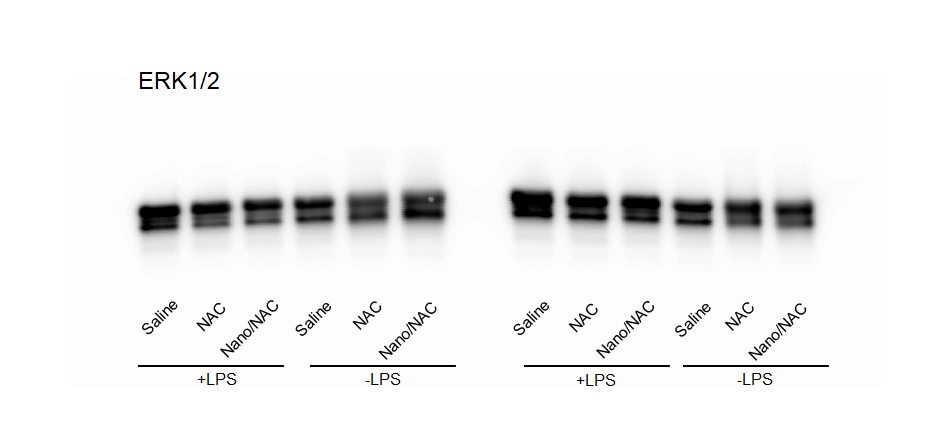


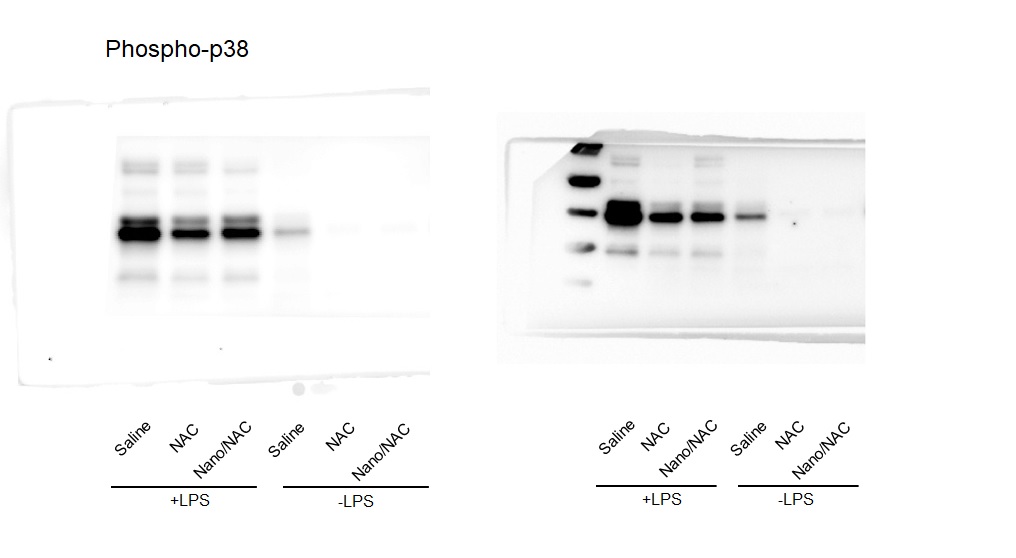


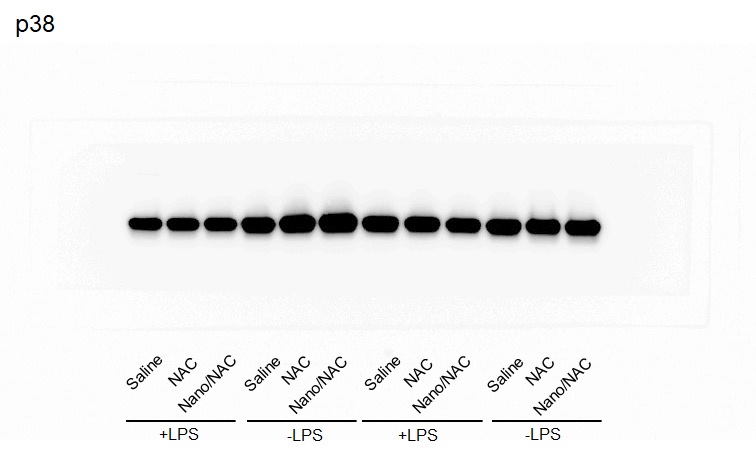


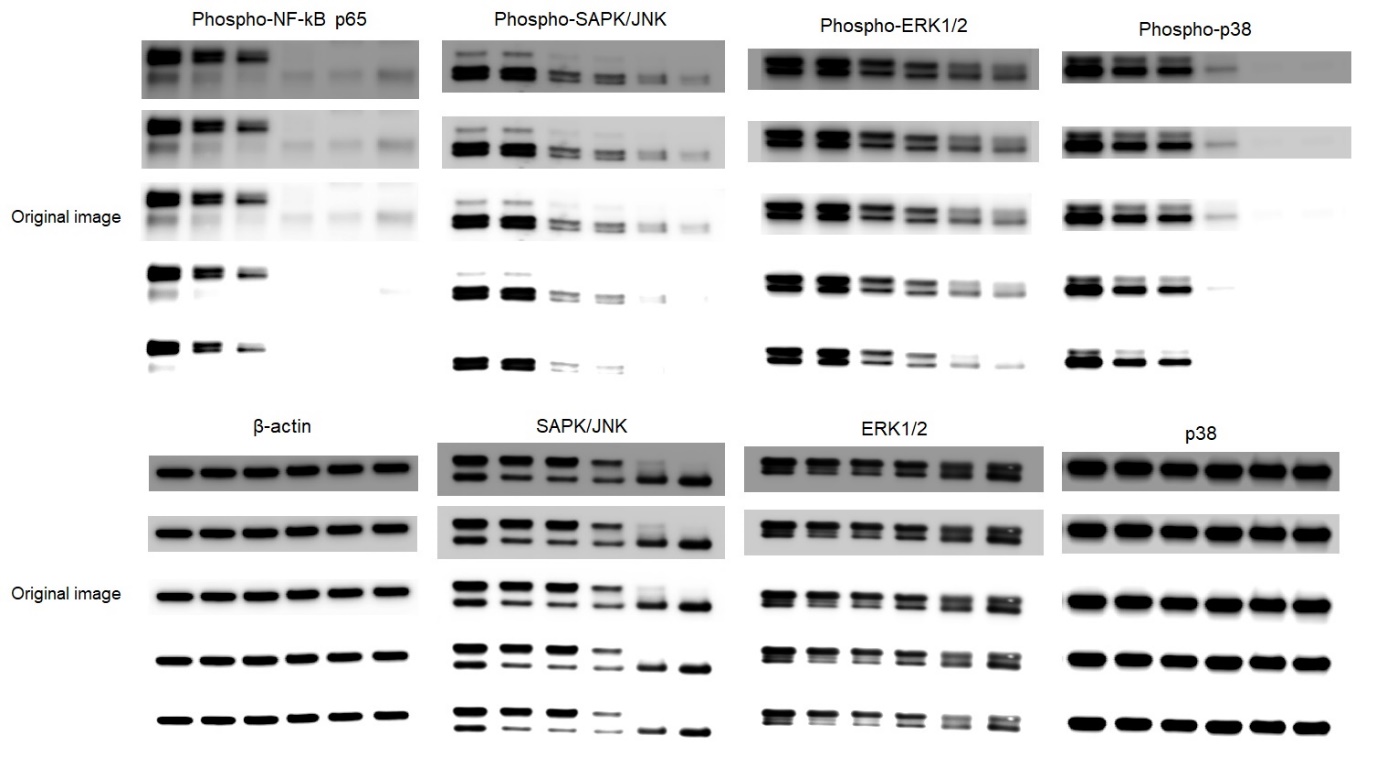

Supplement: Supplementary file 1 — Supplementary Information. [file 41598_2021_1624_MOESM1_ESM.docx]
